# LUCS (Light-Up Cell System), a universal high throughput assay for homeostasis evaluation in live cells

**DOI:** 10.1038/s41598-017-18211-2

**Published:** 2017-12-22

**Authors:** Sylvain Derick, Camille Gironde, Pierre Perio, Karine Reybier, Françoise Nepveu, Alain Jauneau, Christophe Furger

**Affiliations:** 1 Anti Oxidant Power AOP/MH2F-LAAS/CNRS, 7 avenue du Colonel Roche, BP 54200, Toulouse Cedex, 31031 France; 2 Université de Toulouse, PHARMA-DEV, UPS-IRD, 118, route de Narbonne, Toulouse Cedex, 31062 France; 3 CNRS, Institut Fédératif de Recherche 3450, Plateforme Imagerie, Pôle de Biotechnologie Végétale, Castanet-Tolosan, 31326 France

## Abstract

Observations of fluorescent cyanine dye behavior under illumination at 500 nm lead to a novel concept in cell biology allowing the development of a new live cell assay called LUCS, for Light-Up Cell System, measuring homeostasis in live cells. Optimization of the LUCS process resulted in a standardized, straightforward and high throughput assay with applications in toxicity assessment. The mechanisms of the LUCS process were investigated. Electron Paramagnetic Resonance experiments showed that the singlet oxygen and hydroxyl radical are involved downstream of the light effect, presumably leading to deleterious oxidative stress that massively opens access of the dye to its intracellular target. Reversible modulation of LUCS by both verapamil and proton availability indicated that plasma membrane proton/cation antiporters, possibly of the MATE drug efflux transport family, are involved. A mechanistic model is presented. Our data show that intracellular oxidation can be controlled by tuning light energy, opening applications in regulatory purposes, anti-oxidant research, chemotherapy efficacy and dynamic phototherapy strategies.

## Introduction

Explosion of knowledge in cell biology during the past twenty years has led to the integration of live cell assays into the industry. A recent summary^1^ identified many live cell assays dedicated to measurement and assessment but also pointed out that very few have been used for industrial or, more critically, regulatory purposes.

Homeostasis status is the first relevant piece of information that can be extracted from cultured live cells subjected to a condition (i.e. drug candidate, toxic substance, physical stress,…). Change in homeostasis refers to the signs of massive irreversible cell attack leading to apoptosis, necrosis or to major plasma membrane alterations but also to other subtle events, possibly reversible, that modify the cell’s behavior^2^. The main homeostasis signals that have been exploited so far in cell analysis are (i) cell respiration, measured by ATP or NAD(P)H levels^3^, (ii) plasma membrane integrity^4^, (iii) plasma^5^ or mitochondrial membrane potentials^6^, (IV) lysosomal activity, measured through H^+^-ATPase activity^7^ and (v) oxidative stress^8^. The main obstacles to their use in regulatory and industrial applications are the complexity of assay implementation and their inability to be standardized in high throughput or multiplex formats with good robustness (Z’ factor) values.

As a consequence, straightforward assays measuring homeostasis in live cell are urgently needed. This is especially true in the regulatory arena where all OECD-adopted cell based assays, mainly dedicated to toxicity risk assessment, are limited in their throughput and application^9^.

Here, we propose a novel method for live cell assays called LUCS based on the light-induced properties of certain cyanine dyes. While dye fluorescence levels remain low in healthy cells due to drug efflux transport^10^, light induction triggers the generation of intracellular oxidative species^11^ that disrupt cell homeostasis leading to a massive entry of the dye and a consequently huge increase in fluorescence. Conversely, when cells have experienced a prior loss in homeostasis, light induction has no influence on the observed fluorescence levels. The experimental procedure has been optimized to a single addition of the dye in the culture medium followed by an intense flash of light. Cell homeostasis status is deduced from the ratio between post- and pre-fluorescence levels. The process has then been standardized for 96- or 384 well-plates and the Z’ factor has been evaluated. Intracellular events explaining pre- and post-fluorescence levels have been investigated and partially unveiled. The ability to monitor light up and down proved that the assay can analyze subtle changes in cell homeostasis providing an added value over other live cell assays. An example of a regulatory application is given and other industrial applications are discussed.

## Results

### Light-up cell system (LUCS) discovery and optimization

#### Observation of light-induced cell light-up by asymmetrical cyanines

**Figure 1:**
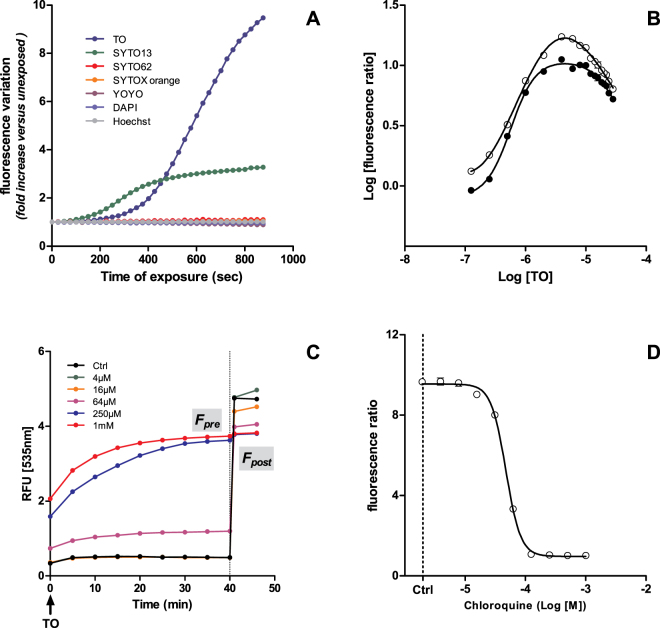
LUCS assay demonstration and optimization on HepG2 cells: (**A**) light-induced fluorescence observed in live cells treated with seven different cell fluorescent dyes (1 h, 4 µM), then illuminated full power with a Varioskan reader up to 900 s; (**B**–**D**) LUCS experiments using thiazole orange (TO): (**B**) TO (1 h, 0.25–32 µM) dose-dependent evolution of LUCS fluorescence ratios (F_post_/F_pre_) with F_post_ measured 1 (dark circles) or 60 min (open circles) after light application; (**C**) fluorescence curves obtained after treatment (24 h) at increasing doses of chloroquine, T = 0: addition of TO 4 µM, T = 40 min, LED illumination (470 nm, 240 mJ/cm^2^); (**D**) chloroquine dose-response curve as measured by LUCS fluorescence ratios. Data presented are representative of 2 (A,B) and 6 (C,D) experiments.

Commercial fluorescence readers are not strong sources of light as their main function is to measure fluorescence level in a way that does not (or does weakly) alter dye excitability or relaxation properties. However, when cultured cells are treated with appropriate dye concentration and submitted to continuous excitation under a commercial fluorescence reader, light-up can be observed. HepG2 cells were cultured in 96-well plates for 24 h, loaded for 1 h with different classical fluorescent cell dyes at 4 µM and submitted to a continuous excitation at full power in a xenon-based Varioskan plate reader (Thermo-Fisher). For each dye, excitation was set up within a 12 nm bandwidth light centered at the maximum excitation wavelength provided by the supplier. Among the six dyes assayed, light-up was limited to SYTO-13 and TO, two asymmetrical cyanines, and started after 2 and 4 min of light exposition, respectively (Fig. 1A). Fluorescence was increased 3 times for SYTO-13 and 10 times for TO after 900 seconds of exposition. Other dyes did not show any evolution in fluorescence. TO and SYTO-13 effects are clearly induced by light as control wells loaded with dye but not exposed to light did not show any difference in fluorescence.

#### Optimization of LUCS process

We then simplified and standardized the LUCS process by exposing TO-preloaded cell samples to a LED source centered at 480 nm. Full light-up was reached right as the flash of light (240 mJ/cm^2^) ended (control curve in Fig. 1C). The process was standardized by measuring fluorescence levels before (F_pre_) and after (F_post_) light application and by calculating the ratio R = F_post_/F_pre_. LUCS amplitude, given by the R value, was evaluated at different TO and SYTO-13 concentrations. R maxima were reached for dye concentrations in the range of 2 to 8 µM. An R value of 10 was reached when F_post_ was measured just after light application (Fig. 1B, black circles) and one of 17 when measured an hour later (Fig. 1B, open circles). A log-log plot shows a bell-shaped fit with R^2^ = 0.99 in both cases.

### Application to cell alteration and acute toxicity evaluation

#### LUCS describes cell homeostasis/alteration status

What makes LUCS interesting for applications is that light-up amplitude varies in a dose-dependent manner according to cell homeostasis/alteration status. This was demonstrated by using HepG2 cells incubated for 24 h with chloroquine, an antimalarial drug known to be cytotoxic, at various concentrations. Results showed that TO F_pre_ varied according to concentration while F_post_ values remained almost unchanged whatever chloroquine condition is applied (Fig. 1C). The 50% efficacy concentration (EC_50_) was evaluated by fitting R ratios with sigmoid dose-response curves. Chloroquine EC_50_ was similar for TO (Fig. 1D) and SYTO-13 (Fig. S1).

#### LUCS is suitable for high-throughput applications

Industrial and regulatory applications need quick, robust and high throughput technologies. As seen in Fig. 1A, LUCS can be performed using commercial fluorescence readers as a source of energy but it would take 15 min to get the result for each experimental condition. As mentioned above, this duration can be strongly shortened (<10 sec) by using an appropriate LED illumination. In order to demonstrate assay suitability to high throughput applications, a simple device based on 24 LEDs each covering illumination of a square of 4 wells of a 96-well plate (alternatively 16 of a 384-well plate) was developed. Robustness was evaluated by statistical analysis using the standard HTS performance Z’ factor. Fourteen 96-well microplates for a total of 1344 wells were treated either with a culture medium or 500 µM of chloroquine for 24 h giving a Z’ factor = 0.80 (Table 1).

**Table 1:** Evaluation of LUCS robustness.

96-well plate Nb	R control	R control SD	R chloroquine	R chloroquine SD	Z’ factor
1	11.16	0.556	1.04	0.015	0.83
2	11.49	0.424	1.06	0.019	0.87
3	11.66	0.540	1.06	0.015	0.84
4	11.55	0.414	1.06	0.015	0.88
5	11.90	0.36	1.07	0.020	0.89
6	11.88	0.53	1.07	0.019	0.87
7	11.64	0.55	1.06	0.015	0.84
8	11.19	0.39	1.04	0.016	0.88
9	11.12	0.45	1.03	0.011	0.86
10	11.27	0.295	1.05	0.013	0.91
11	11.39	0.463	1.05	0.015	0.86
12	11.36	0.303	1.06	0.014	0.91
13	10.40	0.448	1.05	0.017	0.85
14	10.00	0.350	1.03	0.019	0.88
**1–14**	**11.29**	**0.667**	**1.05**	**0.020**	**0.80**

Z’ factors were evaluated as Z’ = 1 − (3 × R_chloroquine_SD + 3 × R_control_ SD)/(R_control_ − R_chloroquine_) with R = F_post_/F_pre_ as defined in text. SD, standard deviation. Z’ factors were calculated for each 96-well plate numbered 1 to 14 and for the 1344 wells together^1–14^.

#### LUCS as a cell acute toxicity assay

**Figure 2:**
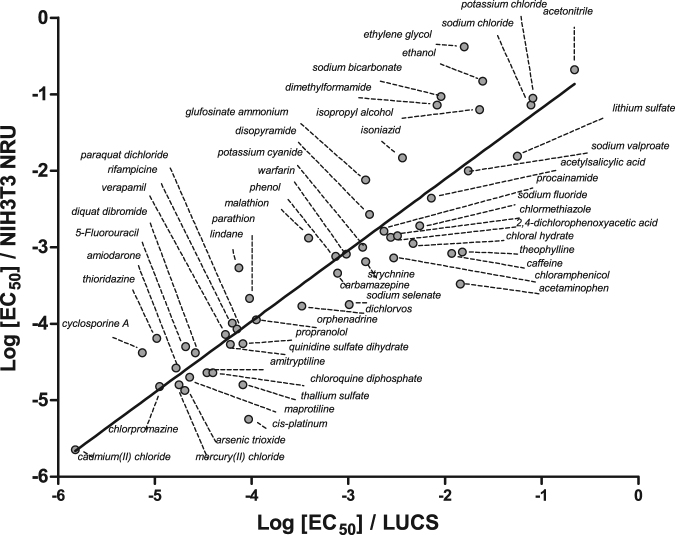
LUCS as a cytotoxicity assay. Regression analysis of 3T3 NRU vs LUCS Log EC_50 _s obtained from dose-response experiments using 53 out of the 97 substances of the ACuteTox European Program databank. Monographs of each dose-response are presented as Supplementary data, see Figure S1.

The LUCS procedure described for chloroquine was applied to 52 other substances taken from the ACuteTox EU program^12^ and covering a large spectrum of origins (drugs, toxins, pesticides, food additives,…). Monographs of the 53 substances assayed by LUCS are given in Fig. S1. Correlation studies were performed by crossing EC_50_ s from HepG2 LUCS and three other state-of-the-art live cell assays, namely (i) Neutral Red Uptake (NRU), which indirectly evaluates intracellular ATP availability by measuring H^+^-ATPase-dependent pH gradient in lysosomes^7,13,14^ (ii) 3-(4,5-di Methyl Thiazol-2-yl)-2,5-diphenyl Tetrazolium bromide (MTT), and (iii) Propidium Iodide (PI)^15^. Data, presented in Table 2, gave R^2^s in the range of 0.75 to 0.92 and a regression line slope always close or equal to 1 whatever cell model was used. An illustration of linear regression analysis is given in Fig. 2 in the case of HepG2 LUCS *vs* 3T3 NRU (n = 53, R^2^ = 0.80, a = 0.94). These results demonstrate that LUCS is relevant for acute toxicity measurement.

**Table 2:** LUCS as a cell acute toxicity assay.

Cell model	Assay	R^2^	a	N	Ref.
3T3	NRU	0.80	0.94	53	^13^ + Fig. 2
NHK	NRU	0.75	0.98	48	^14^
Fa32	NRU	0.80	1.00	51	^14^
3T3	MTT	0.88	1.04	27	^15^
Rat hepatocytes	MTT	0.75	1.00	31*	^15^
HepG2	MTT	0.81	1.05	27	^15^
HepG2	PI	0.92	1.09	16	^15^
SHS-SY5Y	PI	0.83	0.97	25	^15^

Regression analysis of Log EC_50 _s obtained from HepG2 LUCS versus indicated assays using substances of the ACuteTox European Program databank. R^2^, determination coefficient; a, regression line slope; N, number of substances analyzed; NRU, Neutral Red Uptake; MTT, 3-(4,5-di Methyl Thiazol-2-yl)-2,5-diphenyl Tetrazolium bromide; PI, Propidium Iodide; *when malathion, a strong outlier in this series of data, is included: R^2^ = 0.67, a = 0.99, N = 32.

### How does the LUCS assay work?

We then decided to study the mechanism underlying LUCS by considering it as a pre- and post-illumination process. In the pre-illumination step, fluorescence reaches a steady-state level whose value depends on cell homeostasis status. In the case of chloroquine treatment (Fig. 1C), pre-illumination fluorescence (first 40 min) ranges from low to high level according to homeostasis loss. In the post-illumination step, previously low fluorescence levels have increased while high ones remain unchanged. How to explain this peculiar behavior?

#### Steady-state experiments

**Figure 3:**
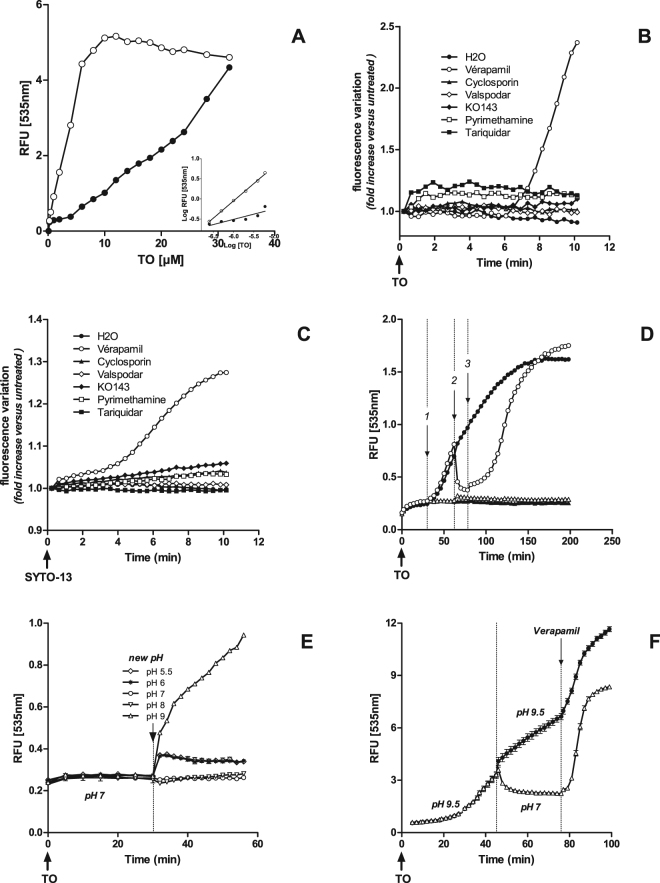
Investigation on pre-illumination stage in HepG2 cells. (**A**) TO dose-response steady state experiments (150 min post TO-labelling) in healthy (dark circles) and chloroquine-treated (1 mM, 24 h, open circles) cells; insert: linear regression analysis based on the six lowest TO concentrations; (**B**,**C**) effect of efflux modulators on fluorescence levels in healthy cells preloaded with TO (**B**) or SYTO-13 (**C**) (2 µM); (**D**) verapamil (200 µM) was added (step 1, T = 30 min post TO 0.5 µM addition, black and open circles), removed (step 2, open circles) and re-added (step 3, open circles); (**E**) cells treated with TO 8 µM were maintained at pH 7, then at various pHs from 5.5 to 9; (**F**) cells treated with TO 0.5 µM were maintained at pH 9.5 (black circles) or at pH 9.5 and pH 7 (open circles), then submitted to 600 µM verapamil. Data presented are representative of 2 (A), 3 (B,C,E,F) or 4 (D) experiments.

Site saturation experiments showed that steady state fluorescence values increased with TO concentration in healthy cells without reaching a maximum (black circles in Fig. 3A). Linear regression analysis (insert, black dots) gave a linear curve with a smaller than normal slope value of a = 0.26 (R^2^ = 0.77) indicating a limitation in marker access to its targets. Steady state fluorescence values in cells treated with chloroquine (1 mM, 24 h) gave a typical saturation curve (open circles in Fig. 3A). Linear regression analysis (insert, open dots) gave a linear curve with a slope value of a = 0.86 (R^2^ = 0.997) close to the normal indicating that the marker accessed its cellular targets with no limitation. Similar results were obtained in a similar independent experiment by replacing chloroquine by HgCl_2_ (32 µM, 4 h) with slope value a = 1.01 (R^2^ = 0.99).

#### ABC/SLC transport activation may explain LUCS pre-illumination step

Low slope values (≪1) obtained in healthy cells oriented us to the hypothesis of a removal of TO and SYTO13 by ABC/SLC efflux systems. Seven selective inhibitors of efflux proteins were assayed. Only verapamil was able to increase fluorescence levels, mimicking the light effect in amplitude. This effect took place after 3 and 6 min in TO (Fig. 3B) and SYTO-13 (Fig. 3C) labeled cells, respectively.

Interestingly, reversion of fluorescence amplitude was observed soon after verapamil was removed from the culture medium (Fig. 3D, stage 2). Verapamil can again exert its effect if re-introduced in the culture medium (stage 3). Eventually, the same fluorescence levels are reached whether verapamil is added at once (dark circles) or in the three-step procedure (open circles).

The strong influence of basic but not acidic pH on TO fluorescence levels is also informative. Fluorescence intensity remained slightly unchanged for at least 45 min at low pH values (5.5 to 8). In contrast, pH 9 and above triggered a very quick (less than 1 min) and massive increase in TO fluorescence level that went on during the next 45 min (Fig. 3E). Again, a return to pH 7 during the process reversed fluorescence intensity (Fig. 3F). In both situations, cells remained reactive to verapamil, even after 75 min.

#### Reactive Oxygen Species (ROSs) are produced during LUCS illumination step

**Figure 4:**
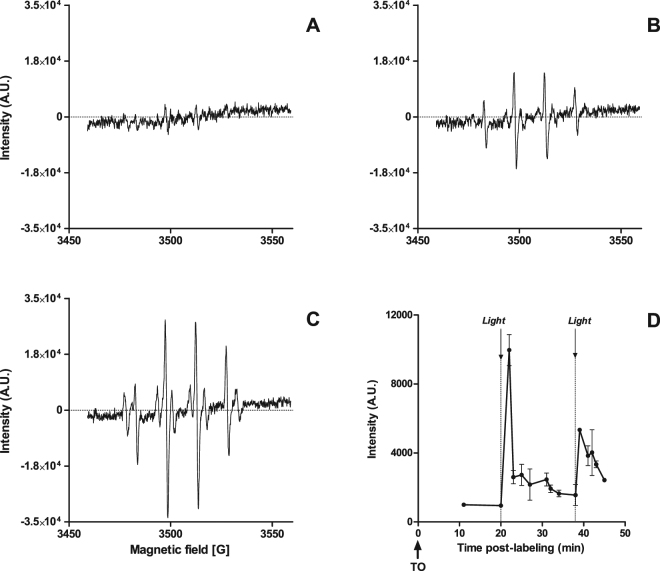
Investigation on post-illumination stage, involvement of reactive oxygen species (ROSs): (**A**–**C**) CHO cells were preloaded with 4 µM TO for 20 min at room temperature, DMPO (50 mM) was added, LED light (240 mJ/cm^2^) was applied (**B**,**C**) or not (**A**) and electronic paramagnetic resonance (EPR) was recorded in the following minute; in (**C**) H_2_O was replaced by D_2_O 20 min before TO was added; (**D**) kinetic RPE follow-up including two successive light applications showing the amplitude of the largest RPE peak (3496 ± 2 gauss).

^•^OH production seems to be linked to singlet oxygen (^1^O_2_) generation since RPE signal intensity was twice higher when H_2_0 was replaced by D_2_0, an environment known to increase ^1^O_2_ lifetime (Fig. 4C). Interestingly, EPR kinetic studies indicated that LUCS triggers a burst of ROS production that vanishes after only two minutes but can be generated again by a new illumination after a latency of a few minutes **(**Fig. 4D **)**. Notably, classical modulators of ROS production such as mannitol, SOD and catalase did not show clear effect on EPR signal produced by LUCS (not shown).

We then performed Electron Paramagnetic Resonance (EPR) spin trapping experiments using 5,5-dimethyl-1-pyrroline-*N*-oxide (DMPO) as a radical trap on CHO live cells to measure extracellular production of ROSs. RPE spectra were recorded before and after LED light application at the usual LUCS intensity and time course. Spectra obtained with growing cells showed little or no ROS production (Fig. 4A) while spectra obtained after LUCS illumination showed the typical quadruplet signature of the DMPO/hydroxyl radical (^•^OH) adduct (Fig. 4B).

## Discussion

The future of an innovative live cell assay clearly relies in its capacity for being universal, simple, high-throughput, adapted to multiplexing and mechanically well understood. Last but not least, it must also reveal information that is not available through other assays.

### Implementation

The LUCS assay is straightforward. TO, or its daughter molecule SYTO-13, is simply added to the culture medium. Fluorescence is read at steady state (reached after 15 min) and again after a flash of light. Analysis is made in the same well with no other variable than the few seconds that have passed between the two measures. Importantly, the F_post_/F_pre_ ratio mode makes the result, to a large extent, independent of the local variables such as the density of cells (chloroquine EC_50_ = 55.06 ± 3.36 µM in the range of 50000 to 150000 cells/well). The LUCS result is robust as confirmed by an inter-plate Z’ value of 0.8 > 0.5, showing applicability to high throughput applications (Table 1).

Simplicity of implementation and robustness may already be claimed by other well established assays such as ATPLite^16^ or Cell Titer Glow^17^ that measure homeostasis status by evaluating cell ATP concentration. However, such approaches require cell lysis and lack the ability to be integrated in more informative multiplex analyses. Label-free approaches based on impedance^18^ or optical^19^ measures are generic and elegant ways to measure homeostasis within cell populations. These techniques are non invasive, very sensitive and can detect very subtle changes in cell homeostasis. However, they are not adapted to multiplexing and need dedicated instruments and consumable items whose cost remains prohibitive.

Another important issue for multiplex analysis is the gain of fluorescence observed in LUCS. Intense light application to live cell fluorescent dyes usually triggers photo-destructive processes that tend to fade the signal. The tendency of LUCS to increase (rather than decrease) the fluorescence level is unique and cannot be mistaken with other signals such as substance intrinsic fluorescence, for instance. This clearly adds value to the LUCS assay.

### LUCS measures a true signal of cell homeostasis

Homeostasis status information produced by LUCS draws the assay closer to classical MTT (or WST-1 advanced protocol) and NRU assays (Table 2). The two cell assays measure homeostasis in relation to cell metabolism, the former by the activity of cytoplasmic and mitochondrial NAD(P)H-dependent oxidoreductases^20^, the latter by the ATP-dependent proton pump activity that keeps a lysosomal/cytoplasmic pH differential leading to discrete neutral red labeling^21^. However, the MTT 1 to 4 hour step of cell lyses and the NRU dependence to image analysis restrict the value of both approaches in terms of multiplex applications. The LUCS assay circumvents these issues by short incubation times and readouts that keep cells operational for parallel or further analyses.

### Investigation on pre-illumination levels of fluorescence

#### Steady-state experiments

If TO photophysical properties have been extensively studied with respect to applications in DNA/RNA quantification, far less is known about its behavior in live cells, apart from topological information^22,23^ (Fig. S2). We studied TO access to intracellular nucleic acids in live cells by steady state experiments. When TO is added to the culture medium, its fluorescence increases up to a plateau that is characteristic of each dye concentration. This heightened fluorescence is known to be due to a drastic change in TO quantum yield which remains extremely low (2 × 10^–4^) in aqueous solution due to free rotation of the two rings around the methine bridge of the molecule^24^. In this situation, relaxation energy is essentially non-radiative, dissipated by internal conversion through an ultrafast intramolecular twisting (100 fs) at the excited state. Conversely, quantum yield can be multiplied by 500 to reach 0.1 when TO binds to double strand DNA^25^. This has been attributed to a restriction in torsion capacity after the dye is intercalated or bound to the minor groove of the DNA^26^.

Our observations on healthy cells show that steady-state fluorescence levels increase with TO concentration but without reaching saturation (Fig. 3A). For low TO concentrations (0.125 to 4 µM), linear regression analyses show slopes from 0.25 to 0.27 (N = 8), far below the normal. On the contrary, steady state fluorescence values obtained after the cell has been altered by a toxic compound reach a maximum for TO ≥8 µM, as expected if we assume that the dye is in excess over the number of available target sites. Slopes obtained for the latter condition rank from 0.86 to 0.97 (N = 3) meaning there is no limitation for TO to reach its cellular targets. These results prompted us to suspect the ABC/SLC drug efflux system to be responsible of the low and non-saturable fluorescence levels observed in healthy cells.

#### Effect of drug efflux modulators

P-glycoprotein (P-gp) does not seem to be involved in TO removal as none of the highly specific P-gp inhibitors such as valspodar (second generation) or tariquidar and zosuquidar (third generation^27^) could influence fluorescence level (Fig. 3B,C). The BCRP family does not seem to be involved either as KO-143 remained without effect. Involvement of the MRP family could not be determined as MK571 showed an intrinsic fluorescence in contact with TO leading to non-conclusive results. The effect of cyclosporine A which is a wide spectrum modulator of ABC efflux transport^28^ was also insignificant. Of interest, only verapamil (≥200 µM) resulted in a quick (within minutes) and robust increase in fluorescence in both TO and SYTO-13 loaded cells. This is not due to a verapamil-induced cytotoxicity as the effect is reversed as soon as the compound is removed (Fig. 3D). Moreover, the reversion is challenged as soon as verapamil is added again suggesting an intimate cell mechanism is at work. Verapamil was first described as a P-gp inhibitor (first generation) but recent studies^29^ indicate that it should not be considered as a blocker but as a competitive inhibitor acting as a substrate of P-gp, a hypothesis confirmed by an X-ray study^30^. These data together with the absence of effect of second and third generations of P-gp blockers seem to eliminate the P-gp efflux family from the LUCS process.

Verapamil is also widely used in clinics in the treatment of atrial arrhythmias where it acts as a Ca^2+^ antagonist drug targeting voltage-gated calcium channels (Ca_v_) activated at hyperpolarized membrane potentials^31^. Recent crystallographic and functional analyses of drugs binding to a bacterial Ca_v_ channel^32^ showed that verapamil blocks calcium flux by physically binding the central cavity of the pore. However, this frequency- and voltage-dependent action of verapamil is totally unexpected when using HepG2 or other classical cell lines in culture dishes.

#### Putative implication of MATE antiporters

Among the five families of multidrug transporters so far identified, only one, the ABC family, is powered by ATP, the four remaining (MATE, MFS, RND and SMR) utilize either H^+^ or Na^+^ electrochemical gradients to export drugs. While RND and SMR families seem to be restricted to bacteria, MFS and MATE are distributed ubiquitously in all kingdoms of living organisms. These two last families are also known to exclusively employ an H^+^ gradient, at least for MATE eukaryotic representatives (SLC47 group)^33^. Both MFS and MATE families are also known for the vast variety of substrates they can flush out of the cell, particularly polyaromatic organic drugs that carry positive charges^34^. Yet, TO and SYTO-13 are small size polyaromatic monovalent organic cations which make them putative substrates of MFS and MATE.

Of most interest is the discovery that verapamil inhibits members of MATE^35^ and MFS^36^ families. The first 3.0 Å resolution X-ray structure of the *Bacillus halodurans* MATE (DinF-BH) in complex with verapamil was recently published^37^. It showed that verapamil occupies the multi-drug binding site preventing drugs from binding the antiporter. More intriguing is the ability of verapamil bound to different MATE channels to adopt different conformations that could explain its flexibility and versatility^38^. The selective verapamil-dependent increase of TO and SYTO-13 fluorescence in LUCS could thus be explained by retention of the dye inside the cell once antiporter activity is blocked.

The antiporter hypothesis was tested by maintaining TO-treated HepG2 cells at different extracellular pH values (Fig. 4A). Results show that there is little or no impact of low pH values (8 down to 5.5). On the contrary, TO fluorescence dramatically increased at pH 9 and above, indicating that proton availability is bound to be involved in TO access to its intracellular targets. At low pH values, proton availability is high and the antiporter exchange is in favor of TO being released out of the cell, explaining the low fluorescence. On the contrary, at high pH values, protons are scarce, TO cannot be removed out of the cell, triggering a huge increase in fluorescence. This explanation is reinforced by the fluorescence being dynamically down regulated after reversion of pH 9.5 towards pH 7 (Fig. 4B).

These results altogether seem to involve cation/H^+^ antiporter in the pre-illumination step of LUCS, even in the absence of effect of pyrimethamine, a putative MATE modulator described as a blocker or the metformin competitive inhibitor^39^. In line with the low selectivity of efflux channels, we cannot exclude that different cell efflux systems (MATE and ABC for instance) are at work at a different level according to the cyanine used and the multi-drug resistance status of the cell.

### Investigation of post-illumination levels of fluorescence

TO bound to DNA or RNA in solution can be self-quenched because of a high local concentration of the compound acting either as an intercalator between base pairs or as a minor groove binder^40^. Following this, one could explain the increase of TO fluorescence by a de-quenching process due to illumination-triggered photobleaching. An example of such de-quenching is given by fluorescein whose fluorescence is increased by laser illumination in liposomes^41^. Alternatively, a direct TO light-induced genotoxic effect could trigger nucleic acid modifications leading to an increase of fluorescence quantum yield. These hypotheses were tested in live cells. Data presented in Figs S3,S4 and Table S1 prove that light-up observed in LUCS is not due to direct photophysical modifications at the level of the dye itself.

The fact that LUCS seems to be restricted to some asymmetrical cyanines (Fig. 1A) can be explained by the highly reactivity that these compounds show at the excited state in comparison to their symmetrical counterparts or other cell dyes^42^. One of the classical pathways to TO relaxation is through energy transfer toward oxygen^43^ producing singlet oxygen as the oxidative source of photoinduction. We then addressed this aspect in live cells and demonstrated by RPE and D_2_0 experiments that LUCS triggers singlet oxygen and downstream radical hydroxyl (^•^OH) production (Fig. 4).

### LUCS “Trojan Horse” mechanistic model

**Figure 5:**
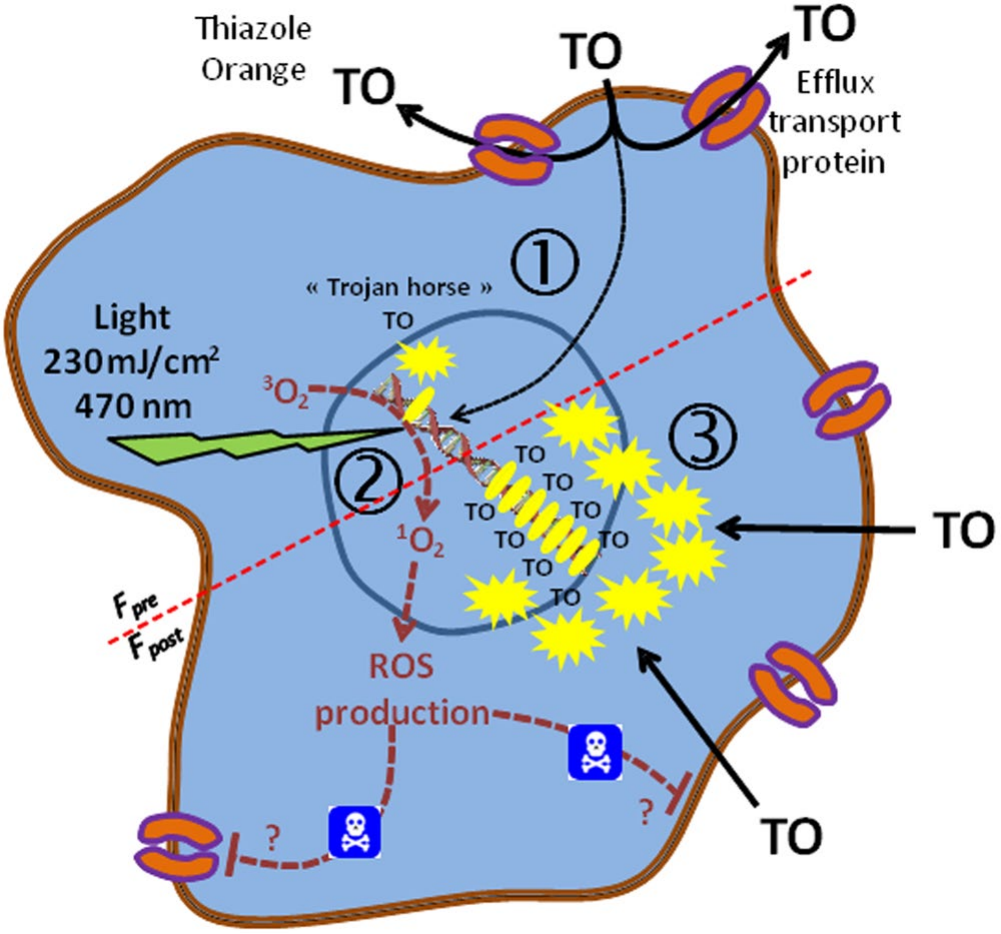
“Trojan horse” mechanistic model of LUCS: **1**, TO is mainly removed out of the cell by efflux transport limiting its access to nucleic acid targets; low fluorescence (F_pre_) is observed; **2**, light is applied inducing ROS production that alters efflux and/or other cell functions; **3**, massive entry of TO triggers increase in fluorescence emission (F_post_).

Together, these data have enabled us to develop a model for the LUCS process presented in Fig. 5. **Step 1**: pH and verapamil’s reversible effects, and the low slope values (0.26) obtained in TO dose-response steady state experiments performed in healthy cells indicate that TO is expelled from living cells by an efflux function. In these conditions, only a small quantity of dye can reach the DNA (alternatively RNA), the TO acting in a similar way as the warriors hidden in the Trojan horse. **Step 2**: RPE data show that light induction of TO produces ROSs, known to produce oxidative damages to intracellular bio-macromolecules^44^. **Step 3**: cellular oxidative stress “opens the gates” (by loss of plasma membrane efflux function or more damaging effects) allowing the massive entry of TO and the subsequent increase of fluorescence.

### Universality of LUCS

Surprisingly, LUCS worked on almost every cell model we assayed so far and was validated on 16 different cells lines or unicellular organisms from prokaryotic and eukaryotic origin (Table S2). This shows how the mechanism at work is shared by most of (if not all) living beings. Nucleic acids targeted by TO are at the very basis of life on earth and the two cellular events deciphered in LUCS, drug efflux on the pre-illumination step and ROS production on the post-illumination step, are also shared by most organisms. This is in accordance with the MATE family being present in most if not all branches of the phylogenetic tree of living beings^45^.

In addition to applications in toxicity evaluation^46^ and risk assessment demonstrated above, the LUCS assay can easily be integrated in methods used in a regulatory context e.g. OECD test guidelines (TG) 431, 432, 439, 491 and 492, and in high throughput screening programs. Taking into account the simplicity of the procedure together with the versatility of the cell lines or unicellular organisms compatible with LUCS, we may envisage its implementation within organs-on-a-chip or mobile devices for detection of pollutants in complex samples.

Knowledge of the mechanism underlying LUCS also should pave the way towards new applications such as research of multidrug resistance modulators or evaluation of chemotherapy efficacy on patient cells prior to treatment. The ability to induce and control intracellular oxidation in live cells could also lead to applications in antioxidant research. Because ROS are associated with deleterious cell effects, LUCS could eventually be used as an alternative to dynamic phototherapy protocols already applied in the treatment of certain cancers.

## Methods

### Material and reagents

Thiazole orange (TO), verapamil, cyclosporin, KO143, HEPES, HCl, NaOH, and the 53 chemicals taken from the AcuteTOX EU program (see Table S1 for details) were purchased from Sigma-Aldrich (Saint-Quentin Fallavier, France). Tariquidar and Valspodar, were purchased from MedChemTronica (Stockholm, Sweden). SYTO13, SYTO62, YOYO-1, SYTOX orange, Hoechst, DAPI, Deuterium oxide (D_2_O), Gibco DMEM (high glucose, GlutaMAX supplement and pyruvate), fetal bovin serum (FBS) (HyClone), pen-strep solution (100X) (Gibco), 0.05% Trypsin-EDTA (HyClone), Gibco DPBS without Calcium and Magnesium (1X) and HyClone Ham’s Nutriment Mixture F12 Media were purchased from Thermo Fisher Scientific. 5,5-Dimethyl-1-Pyrroline-N-Oxide (DMPO) was purchased from Dojindo Molecular Technologies (Rockville, MD, USA). HepG2 and CHO-K1 cell lines were purchased from the American Type Cell Collection (ATCC) (LGC Standards, Molsheim, France), catalog number HB8065 and CRL9618 respectively.

### Cell culture

HepG2 cells (passage 15 to 35) were cultured at 37 °C/5% CO_2_ in DMEM medium complemented with 10% FBS and 1% pen-strep solution. Cells were grown up to 70–80% confluence then transferred in clear bottom 96-well microplates at a density of 10^6^ cells/ml (75 µL, 75000 cells/well) and cultured in the same culture medium for 24 h.

CHO-K1 cells (passage 15 to 35) used for EPR experiments were grown up to 80–90% confluence in F-12 medium complemented with 10% FBS and 1% pen-strep solution then detached and pooled at a density of 10^6^ cells/ml in PBS buffer.

### Treatment

Compound treatment or cell dye labelling were performed in serum-free medium to avoid interaction with serum components. Compounds and dyes were added by replacing media or, in the case of the efflux modulator study, by automatic injection. In multistep experiments involving washes (pH and verapamil effect study), TO concentration was always maintained at initial value.

For dose-response experiments, solvent concentration was always ≤1% (vol/vol) and maintained at this concentration in lower doses.

Efflux modulators were added or injected at 50 µM final concentration, apart from verapamil, 400 µM, and pyrimethamine, 200 µM.

For the pH study, HEPES was added to the medium at 25 mM final concentration before experiment. The pH was adjusted at the desired values by addition of HCl (0.2 M) or NaOH (0.2 M) solutions.

### Standard LUCS procedure

HepG2 cells plated in 96-well microplates were incubated for 1 h at room temperature with 4 µM TO. The fluorescence (F_pre_) was measured using a Varioskan Flash Spectral Scanning Multimode Reader (Thermo Fisher Scientific, Waltham, Mass., USA) set up at 505/535 nm (excitation/emission wavelengths). Microplates were then placed in a dedicated illuminator (24 LEDs, 470 nm, each LED centered on the intersection of 4 wells) and illuminated (10 sec, 24 mW/cm^2^). The fluorescence was measured a second time (F_post_) immediately after illumination. This standard procedure was used to evaluate the robustness factor Z’. For other LUCS experiments, TO or SYTO incubation times and concentrations are specified in the legends of the corresponding figures.

### EC50 and determination coefficient (R2) evaluation

For dose-response experiments, F_post_/F_pre_ ratios obtained for each dose were fitted with a mathematical non linear regression model: *Y* = *Bottom* + *(Top-Bottom)/(1* + 10^((LogEC50-X)*HillSlope)), using GraphPad Prism software. 50% efficacy concentration (EC_50_) and *r* ^2^ values were deduced from the regression model.

### EPR analysis

EPR spectra were obtained at X-band and at room temperature on a Bruker EMX-8/2.7 (9.86 GHz) equipped with a high-sensitivity cavity (4119/HS 0205) and a gaussmeter (Bruker, Wissembourg, France). A flat quartz cell (FZKI160–5 × 0.3 mm, Magnettech, Berlin, Germany) was used to measure radical formation. WINEPR and SIMFONIA software (Bruker, Wissembourg, France) were used for data processing and spectrum computer simulation. Typical scanning parameters were: scan rate, 1.2 G/s; scan number, 5; modulation amplitude, 1 G; modulation frequency, 100 kHz; microwave power, 20 mW; sweep width, 100 G; sweep time, 83.88 s; time constant, 40.96 ms; magnetic field 3460–3560 G.

EPR experiments were carried out using the spin-trapping agent DMPO (5,5-dimethyl-1-pyrroline N-oxide). The analyses were performed by adding DMPO (final concentration 50 mM) on 10^6^ CHO cells in PBS buffer. When needed, H_2_O contained in the PBS buffer was replaced by D_2_0. The illumination was performed directly though the quartz flat cell containing the sample.

### Confocal microscopy

Confocal microscopy was performed using an upright microscope (Leica DMRX) equipped with a laser scanning confocal head (TCS Leica SP2 AOBS). Images were acquired using a 60x oil immersion lens (N.A.1.3). The ray line at 488 nm of an argon laser was used for excitation, the emitted fluorescence being collected between 500 and 550 nm.

### Fluorescence lifetime imaging microscopy (FLIM)

A complete description of the FLIM system used here is reported in^47^. Briefly FLIM measurements were performed using a mode-locked Ti:sapphire laser (Tsunami, model 3941, Spectra-Physics, USA) pumped by a 10 W diode laser (Millennia Pro, Spectra-Physics), delivering ultrafast femtosecond pulses of light with a fundamental frequency of 80 MHz. A pulse picker (model 3980, Spectra-Physics) was used to reduce the repetition rate to 2 MHz to satisfy the requirements of the triggering unit (working at 2 MHz). All the experiments were carried out at λ = 850 nm. Images were acquired with a 60x oil immersion lens (Plan Apo 1.4 NA, IR) mounted on an inverted microscope (Eclipse E2000E, Nikon, Japan) coupled to the FLIM system. In this setup the femtosecond-pulsed laser light is scanned into the left camera port of the microscope via a dichroic mirror (λ < 750 nm). The fluorescence emission is directed back into the detection unit through the same camera port. The FLIM unit is composed of two galvo mirrors, scanning along the x and y axes, a relay lens and the streak camera (Streakscope C4334, Hamamatsu Photonics, Japan) coupled to a fast and high-sensitivity CCD camera (model C8800-53C, Hamamatsu). Depending on the expected lifetime of the fluorophores, fluorescence decays were recorded during a time range of 20 ns.

For each image, fluorescence decay profiles from the different cell compartments were plotted and the mean lifetime estimated by fitting data with an exponential function using a nonlinear least-squares estimation procedure. The manual’s user of the software can be freely uploaded http://trigenotoul.com/ressources/imagerie-cellulaire-tissulaire-*in-vivo*/flim/. Statistics were performed with Student’s t-test.

### Data availability

The datasets generated during and analyzed as part of the current study are available from the corresponding author on reasonable request.

## Electronic supplementary material

Supplementary data
