# Supplementary material for: LUCS (Light-Up Cell System), a universal high throughput assay for homeostasis evaluation in live cells
Source: Sci Rep. 2017 Dec 22;7:18069. doi: 10.1038/s41598-017-18211-2 (PMC5741755; doi:10.1038/s41598-017-18211-2)
Supplement: Supplementary file 1 — Supplementary data [file 41598_2017_18211_MOESM1_ESM.pdf]

Supplementary data

LUCS (Light-Up Cell System),
a universal high throughput assay for homeostasis evaluation in live cells

DERICK Sylvain, GIRONDE Camille, PERIO Pierre, REYBIER Karine, NEPVEU Françoise, JAUNEAU Alain and FURGER Christophe

Details on five aspects of the study are given:

1) Monographs (**figure S1**) and EC_{50}s (**table S1**) of the 53 dose-response experiments used to demonstrate acute toxicity application (**figure 2**),

2) The intracellular labelling topology of thiazole orange (TO) dye (**figure S2**) observed in HepG2 live cells,

3) Experimental arguments that allowed to discarding a “dequenching” process as being involved in the fluorescence increase observed in LUCS assay. Based on fluorescence theory, thiazole orange (TO) fluorescence could be self quenched by the proximity of the molecules acting as intercalators within DNA and/or RNA. According to this hypothesis, the progressive light-induced bleaching of the fluorophores could restore fluorescence by dequenching the remaining intact fluorophores. This assumption was discarded by fluorescence life time measurements (**figure S3, table S2**).

4) Experimental arguments that allowed to discarding a modification of dye quantum yield as being involved in the fluorescence increase observed in LUCS assay. In a double labelling experiment where SYTO13 and SYTO62 dyes were sequentially added to cells, LUCS was observed in conditions where SYTO62 had not been in contact with light (i.e. after light induction had ended) (**figure S4**) showing that SYTO62 fluorescence increase can not be explained by modifications of its photophysical properties.

5) A list of the different live models on which LUCS has been observed (**table S3**)

Figure S1

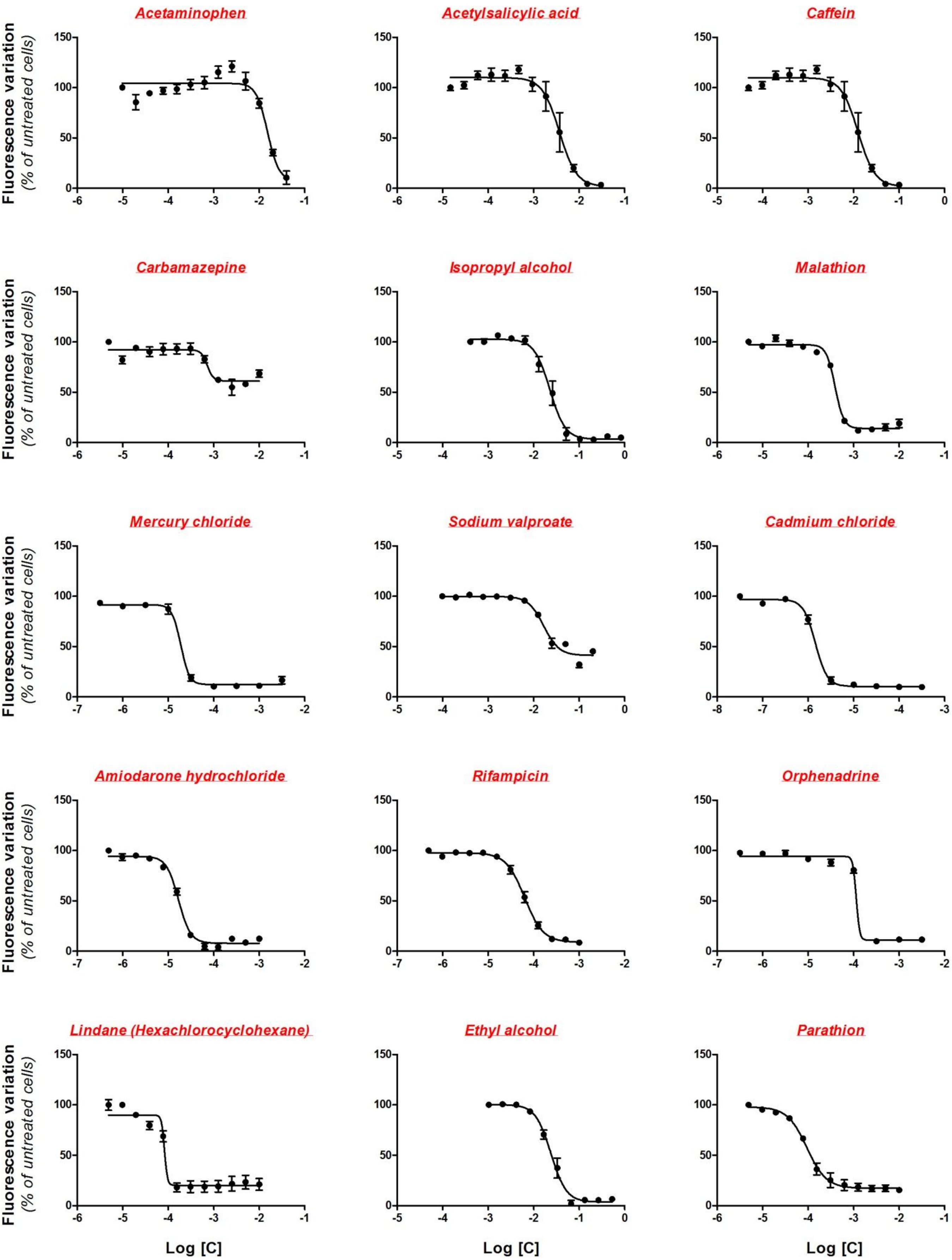
Acetaminophen
Acetylsalicylic acid
Caffein
Carbamazepine
Isopropyl alcohol
Malathion
Mercury chloride
Sodium valproate
Cadmium chloride
Amiodarone hydrochloride
Rifampicin
Orphenadrine
Lindane (Hexachlorocyclohexane)
Ethyl alcohol
Parathion
Fluorescence variation
(% of untreated cells)
Log [C]

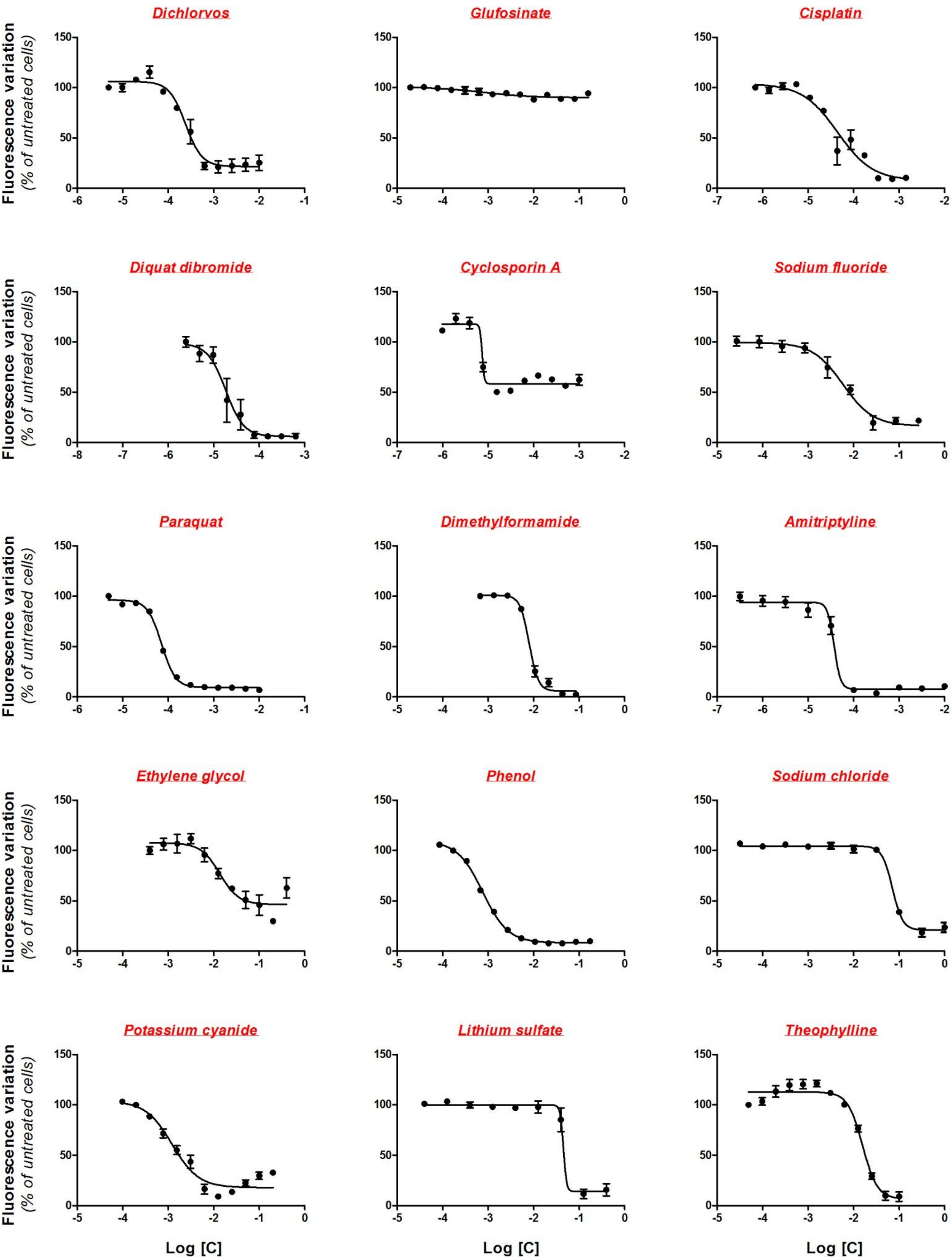
Dichlorvos
Glufosinate
Cisplatin
Diquat dibromide
Cyclosporin A
Sodium fluoride
Paraquat
Dimethylformamide
Amitriptyline
Ethylene glycol
Phenol
Sodium chloride
Potassium cyanide
Lithium sulfate
Theophylline
Fluorescence variation
(% of untreated cells)
Log [C]

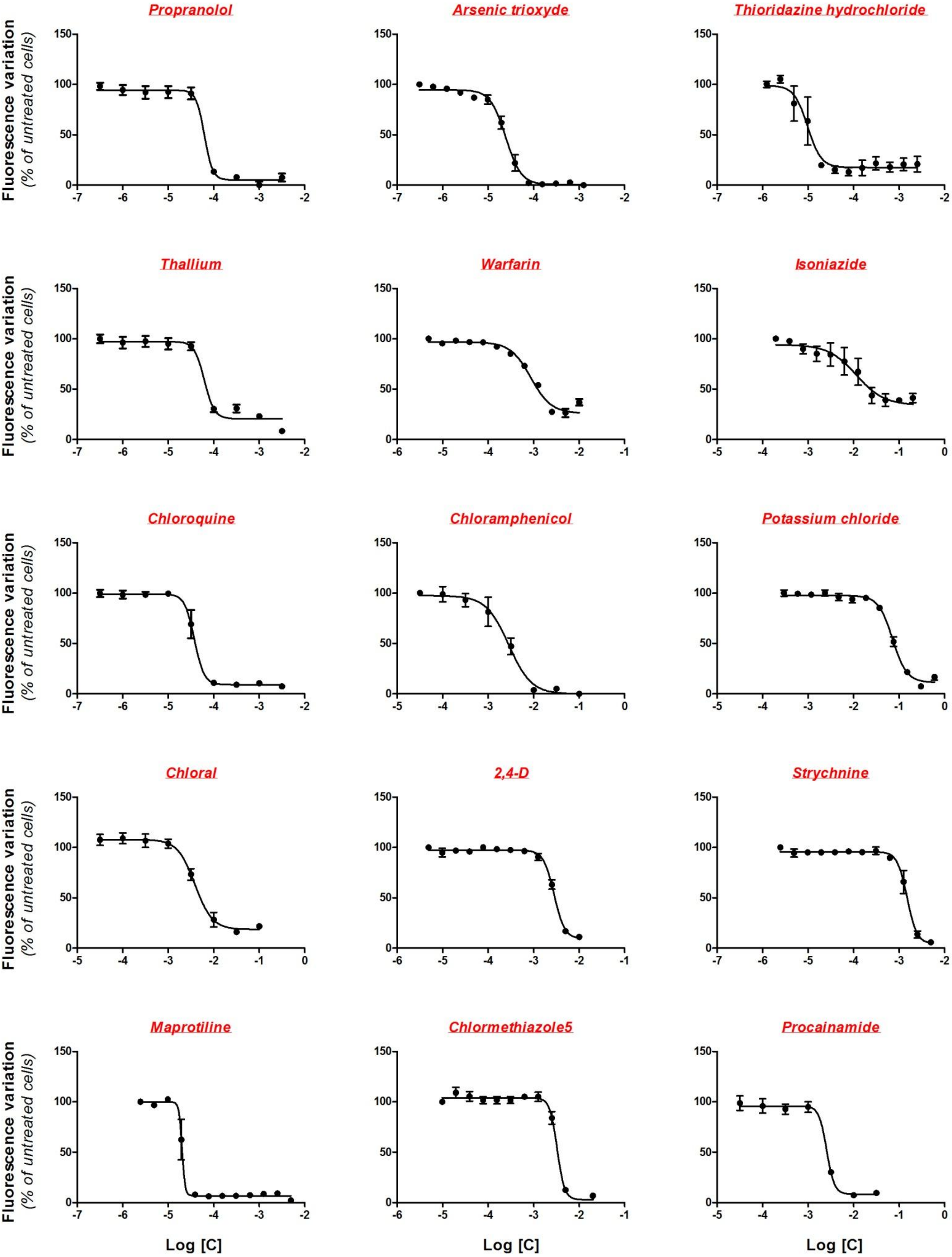
Propranolol
Arsenic trioxyde
Thioridazine hydrochloride
Thallium
Warfarin
Isoniazide
Chloroquine
Chloramphenicol
Potassium chloride
Chloral
2,4-D
Strychnine
Maprotiline
Chlormethiazole5
Procainamide
Fluorescence variation
(% of untreated cells)
150
100
50
0
-7
-6
-5
-4
-3
-2
-1
0
Log [C]

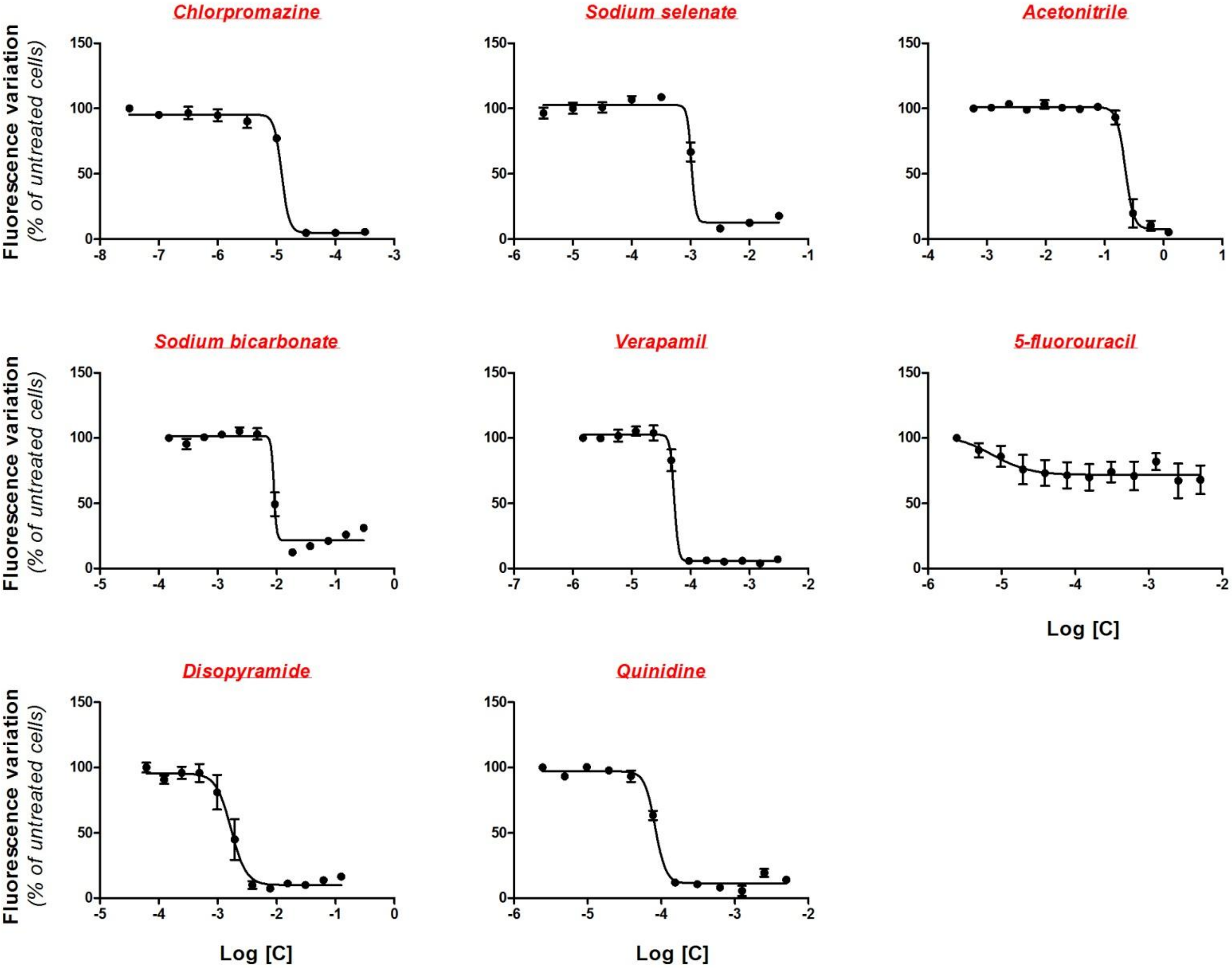

Figure S1. **LUCS assay dose-responses of the 53 compounds used for the regression analysis study (figure 2)**. HepG2 cells cultured in 96-well plates were treated with compounds (53 out of the 67 substances of the ACuteTox European Program databank, according to commercial availability) for 24h. Cells were then treated with SYTO13 at 4µM final concentration for 30 min followed by a fluorescence measurement (F_{pre}) at 520nm. Plates were then exposed to a LED based light source (0.24 J/cm^2 centered at 470nm) followed by a second fluorescence reading (F_{post}). Dots represent $R=F_{post}/F_{pre}$ values normalized to untreated (control) cells. Corresponding EC_{50}s are given in table S1. All experiments were performed at least 3 times and in each case in triplicate. Dots represent means of the 3 experiments (error bars= SD values)

Table S1

Chemical (A-Z)	EC50 (M)	EC50 (log M)
2,4-D	2,77E-03	-2,56
5-fluorouracil	2,08E-05	-4,68
Acétaminophen	1,45E-02	-1,84
Acétonitrile	2,17E-01	-0,66
Acide acétylsalicylique	7,32E-03	-2,14
Amiodarone hydrochloride	1,66E-05	-4,78
Amitryptiline	3,47E-05	-4,46
Arsenic trioxyde	2,05E-05	-4,69
Bicarbonate de sodium	9,17E-03	-2,04
Caféine	1,18E-02	-1,93
Carbamazépine	7,69E-04	-3,11
Chloral	4,64E-03	-2,33
Chloramphénicol	2,92E-03	-2,53
Chlorméthiazole	3,27E-03	-2,49
Chloroquine	3,97E-05	-4,40
Chlorpromazine	1,12E-05	-4,95
Chlorure de Cadmium	1,50E-06	-5,82
Chlorure de mercure	1,80E-05	-4,75
Chlorure de potassium	8,19E-02	-1,09
Chlorure de sodium	7,79E-02	-1,11
Cis Platine	9,32E-05	-4,03
Cyanure de potassium	1,40E-03	-2,85
Cyclosporine A	7,40E-06	-5,13
Dichlorvos	3,31E-04	-3,48
Diméthyl formamide	8,28E-03	-2,08
Diquat dibromide	2,63E-05	-4,58
Disopyramide	1,65E-03	-2,78
Ethanol	2,48E-02	-1,61
Ethylène glycol	1,57E-02	-1,80
Fluorure de sodium	5,53E-03	-2,26
Glufosinate ammonium	1,50E-03	-2,82
Isoniazid	3,61E-03	-2,44
Isopropanol	2,30E-02	-1,64
Lindane	7,44E-05	-4,13
Malathion	3,87E-04	-3,41
Maprotiline	2,28E-05	-4,64
Orphénadrine	1,12E-04	-3,95
Paraquat	7,10E-05	-4,15
Parathion	9,48E-05	-4,02
Phénol	7,45E-04	-3,13
Procaïnamide	2,33E-03	-2,63
Propranolol	5,97E-05	-4,22
Quinidine	8,19E-05	-4,09
Rifampicine	6,38E-05	-4,20
Sodium Selenate	1,02E-03	-2,99
Sodium Valproate	1,76E-02	-1,76
Strychnine	1,51E-03	-2,82
Sulfate de lithium	5,62E-02	-1,25
Thallium	8,10E-05	-4,09
Théophyline	1,51E-02	-1,82
Thiorizadine	1,05E-05	-4,98
Vérapamil	5,36E-05	-4,27
Warfarin	9,57E-04	-3,02

Table S1. EC_{50} of the 56 chemical tested on HepG2 cells with the LUCS assay.

Figure S2

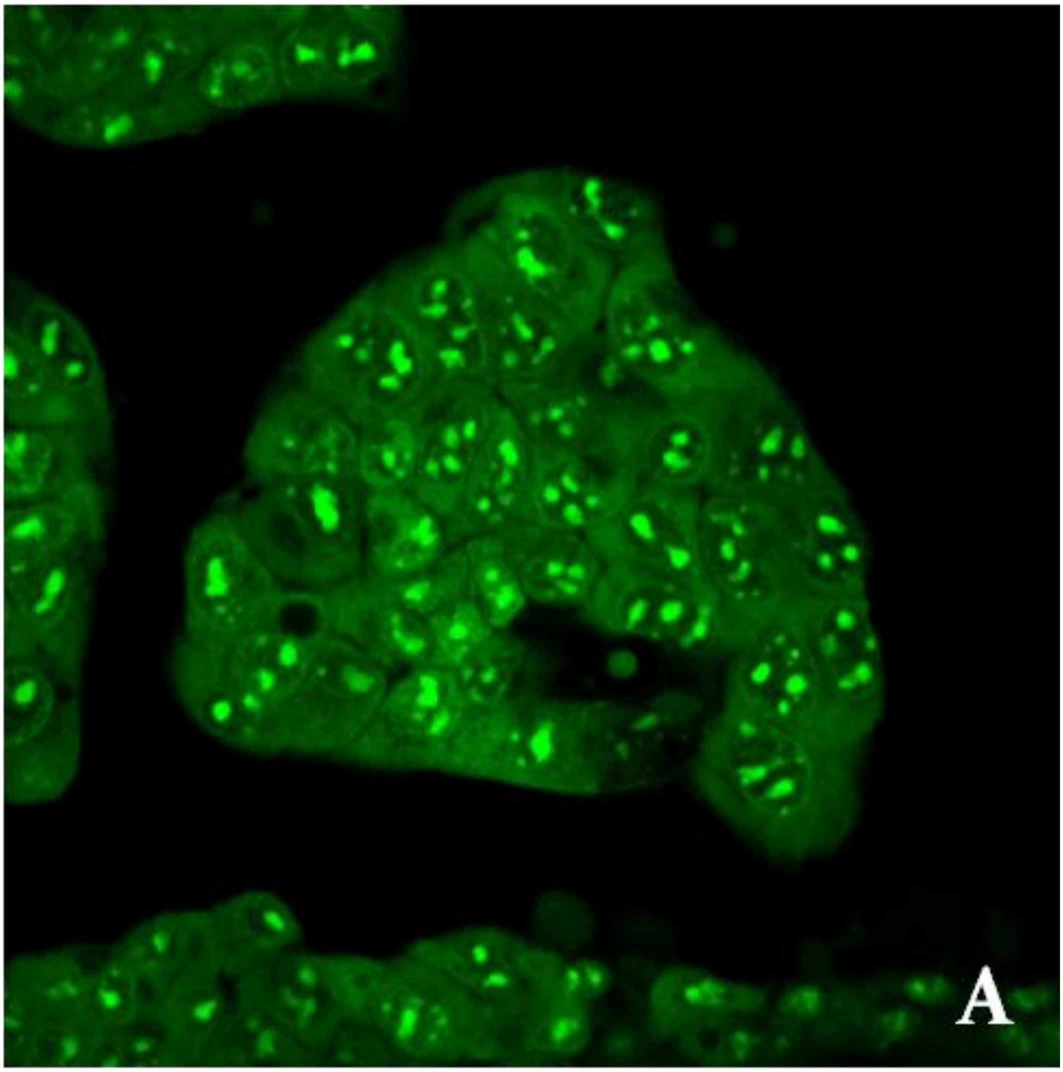

Exc. = 514 nm ; 530 nm < Em. < 560 nm

Figure S2. TO binding topology in HepG2 live cells observed by confocal microscopy at indicated excitation wave lengths and emission windows. As seen by others (see references 10, 11), labeling is predominant in nucleolar-like bodies within the nucleus, in the nucleus and, to a lesser extent, in the cytoplasm.

Figure S3

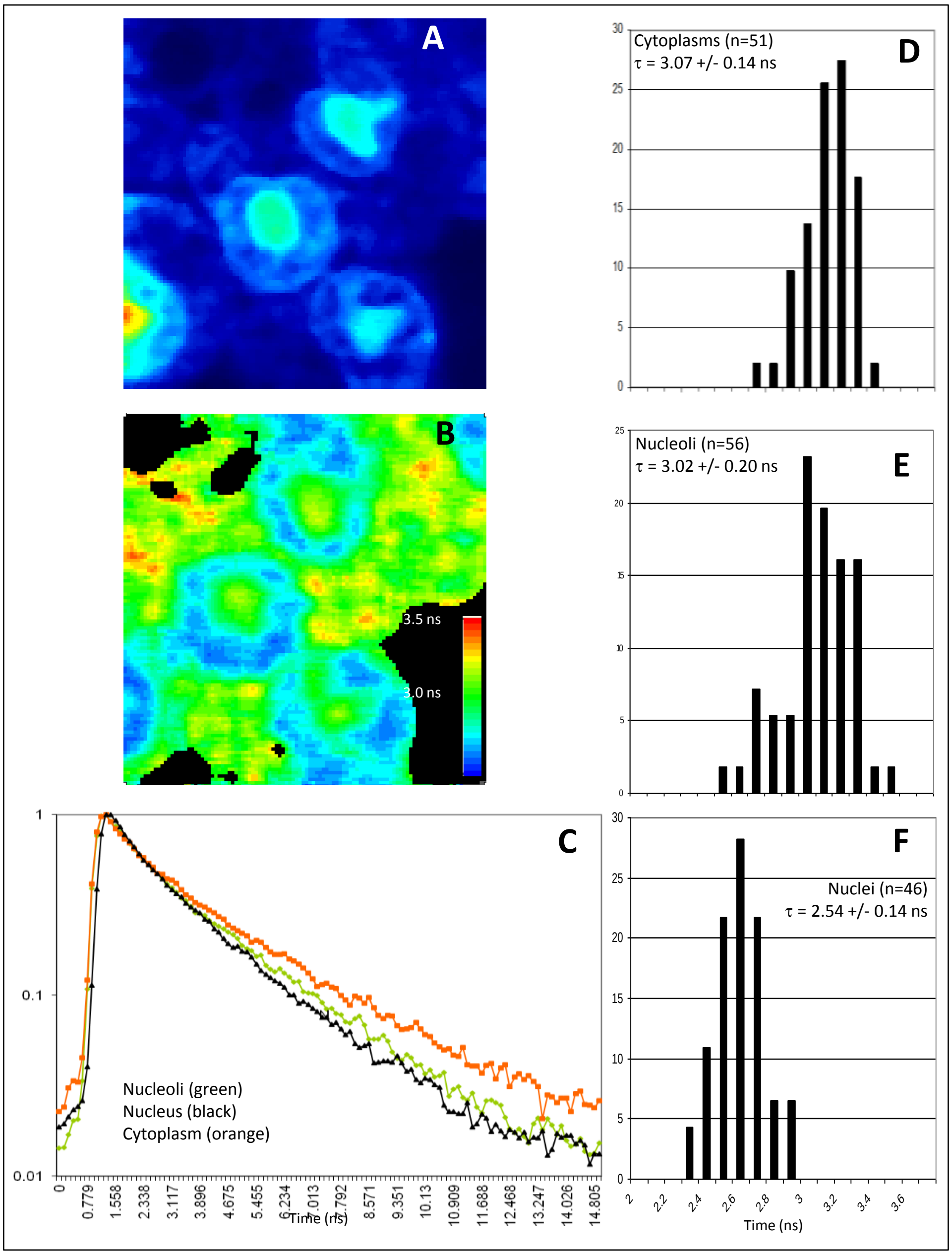

Figure S3. Time-resolved fluorescence spectroscopy in TO-treated HepG2 live cells. Results indicate that excited state TO life-times (τ) vary according to cell compartments: nuclei < nucleola-like bodies < cytoplasm. **A**, cell pattern distribution of fluorescence intensities (bi-photonic excitation, 850 nm); **B**, life-time values (τ) in the same field; **C**, time resolved-fluorescence decays in different HepG2 cell compartments; **D-F**, statistical repartition of τ values according to cell compartments.

Table S2

Cell compartment	Illumination 440 nm	τ medium n sec	SD	SEM	NB	Delta p sec	p-value
Nucleus	-	2.375	0.165	0.028	36	-	-
	+	2.032	0.155	0.021	58	343	$9.5\ 10^{-17}$
Cytoplasm	-	2.992	0.181	0.026	49	-	-
	+	2.850	0.140	0.020	47	142	$4.6\ 10^{-5}$

Table S2. Thiazole orange lifetime values (τ) before and after light application (440 nm) in live HepG2 cells. According to the fluorescence theory, a dequenching process (increase of photon emission following the loss of resonance energy transfer or other quenching processes) should increase fluorescence lifetimes values (τ). We show here that τ values tend to decrease after light application suggesting that TO self-dequenching process is not involved in LUCS assay.

Figure S4

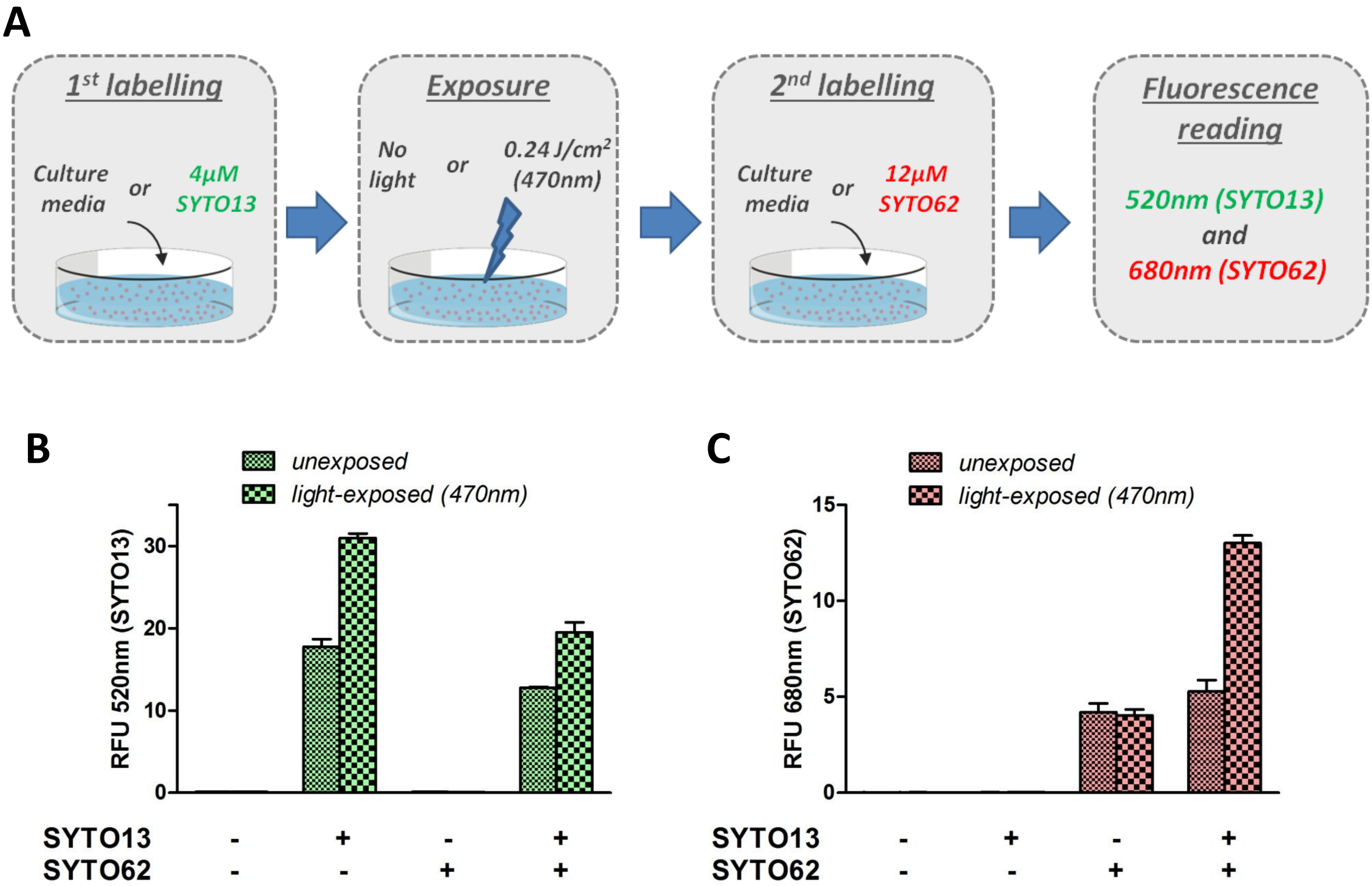

Figure S4. Double labelling experiment. **A.** HepG2 cells were treated for 1h with SYTO13 at 4µM final concentration then exposed (or not) to a LED light source (470nm; 0.24 J/cm^2). SYTO62 was (or not) added at a final concentration of 12µM and then, fluorescence of SYTO13 and SYTO62 was read at 520nm or 680nm respectively. **B. Fluorescence reading at SYTO13 wavelength (520 nm)**. As expected, in cells labeled with SYTO13 only, light exposure at 470nm triggered an increase of fluorescence emission at 520nm as compared to unexposed cells. After SYTO62 addition, fluorescence of both exposed and unexposed cells were slightly reduced, possibly due to a site competition between the two SYTO dyes on nucleic acids. **C. Fluorescence reading at SYTO16 wavelength (680 nm)**. Without light exposure the presence of SYTO13 does not affect SYTO62 fluorescence. However, light exposure at SYTO13 wavelength triggered SYTO62 fluorescence increase. As the SYTO62 dye was added after light exposure, its fluorescence increase cannot be explained by a photo-induced process. This demonstrates that the fluorescence increase associated to LUCS process is not due to modification of intrinsic photophysical dye properties but rather to a massive entry of the dye triggered by a photo-induced process.

Table S3

**Kingdom**	**Class**	**Order**	**Species**	**Cell model description**
Bacteria	γ-proteobacteria	Enterobacteria	*Escherichia coli*	
Chromalveolata	Bacillariophyta (diatoms)			
Plants	Magnolioprida	Solanales	*Nicotiana tabacum*	***BY2*** tobacco cells
Fungi	Basidiomycota	Sporidiales	*Rhodotorula sp.*	
	Saccharomycetes	Saccharomycetales	*Saccharomyces cerevisiae*	
			Saccharomyces boulardii	
Animalia	Actinopterygii	Cyprinodontiformes	*Fundulus notatus*	***PLHC-1***: liver cells from blackstripe topminnow
		Salmoniformes	*Oncorhynchus mykiss*	**RT-GillW1**: epithelial cells from rainbow trout
	Mammalia	Rodentia	*Mus musculus*	**Neuro-2a**: mouse cells from neuroblastoma
			Cricetulus griseus	***CHO*** : cells derived from ovary of Chinese hamster
		Primates	*Cercopithecus aethiops*	***COS7***: fibroblast-like cell line derived from kidney tissue
			Homo sapiens	***THP1***: cell line from an acute monocytic leukemia
				HACAT: immortal human keratinocytes
				**SH-SY5Y** : bone marrow cells from neuroblastoma
				**HeLa**: cell line established from cervical tumour
				**CaCo2**: epithelial hypertetraploid cells from colon
				**HepG2**: cells from hepatocellular carcinoma

Table S3. The 16 prokaryotic and eukaryotic unicellular organisms or cell lines on which LUCS process was evaluated with success
